# Supplementary material for: Orange-Peel-Derived Nanobiochar for Targeted Cancer Therapy
Source: Pharmaceutics. 2022 Oct 21;14(10):2249. doi: 10.3390/pharmaceutics14102249 (PMC9607014; doi:10.3390/pharmaceutics14102249)
Supplement: Supplementary file 1 [file pharmaceutics-14-02249-s001.zip › pharmaceutics-1936622-supplementary.pdf]

Electronic Supplementary Information

to

Orange peel derived nanobiochar for targeted cancer therapy

Daniela Iannazzo,[1*] Consuelo Celesti,[1,2*] Claudia Espro,[1] Angelo Ferlazzo,[1] Salvatore V. Giofrè,[3] Mario Scuderi,[4] Silvia Scalese,[4] Bartolo Gabriele,[5] Raffaella Mancuso,[5] Ida Ziccarelli,[5] Giuseppa Visalli,[6] Angela Di Pietro[6]

[1] *Department of Engineering, University of Messina, Contrada Di Dio, I-98166 Messina, Italy;*
[2] *Department of Clinical and Experimental Medicine, University of Messina, Via Consolare Valeria, I-98125 Messina, Italy*
[3] *Department of Chemical, Biological, Pharmaceutical and Environmental Sciences, University of Messina, Viale F. Stagno d'Alcontres, 98166 Messina, Italy*
[4] *Institute for Microelectronics and Microsystems, National Research Council (CNR-IMM), Ottava Strada n.5, I-95121 Catania, Italy*
[5] *Laboratory of Industrial and Synthetic Organic Chemistry (LISOC), Department of Chemistry and Chemical Technologies, University of Calabria, Via Pietro Bucci 12/C, 87036 Arcavacata di Rende (CS), Italy*
[6] *Department of Biomedical and Dental Sciences and Morphological and Functional Images, University Hospital of Messina, Via Consolare Valeria, 1, 98100 Messina, Italy*
* *Correspondence: diannazzo@unime.it; ccelesti@unime.it*

Contents

Figure S1. Representative CLSM image of A549 cells treated for 24 h with NBC-B.................S2

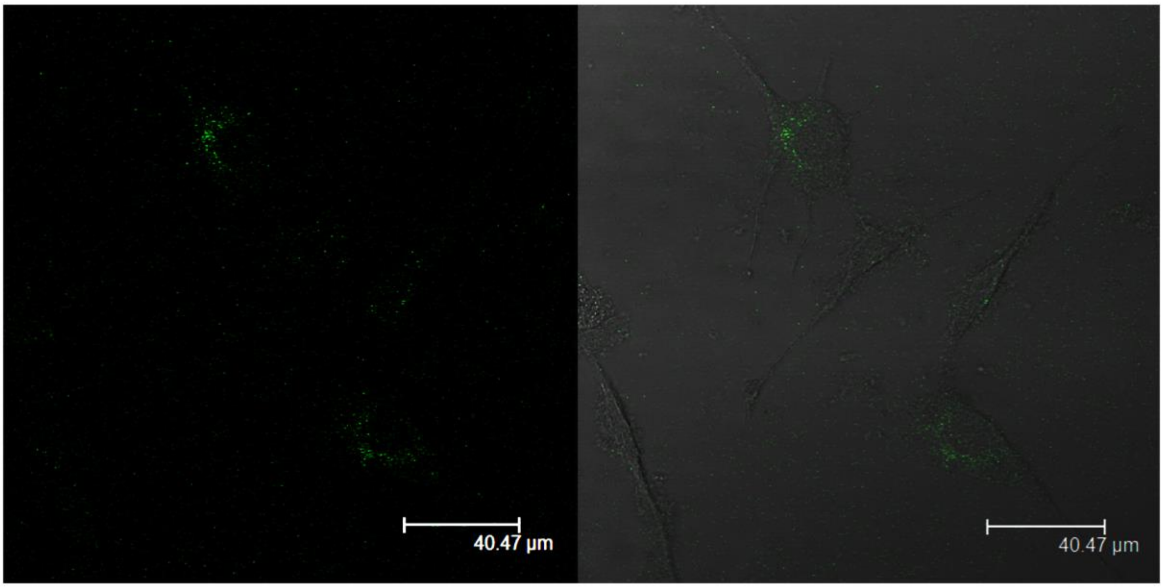

Figure S1. Representative CLSM image of A549 cells treated for 24 h with NBC-B to assess the cell uptake.
